# Making deep immunophenotyping accessible: the successful application of a guided 23-parameter mouse immunophenotyping panel package provided through a shared resource

**DOI:** 10.3389/fimmu.2025.1668405

**Published:** 2026-01-09

**Authors:** Madison G. Isbell, Alex Wendling, Xinyan Pei, Amit Kumar, Padmanabhan Mannangatti, Bradley A. Krisanits, Stanley Cheatham, Marie Michenkova, Kirill Shumilov, Rachel G. Mendoza, Matthew E. Fernandez, Allyn Bryan, Thuy-An Nguyen, Lauren May, Swadesh K. Das, Victoria J. Findlay, Hamid I. Akbarali, Maria Garcia-Bonilla, David D. Limbrick, Douglas H. Sweet, Sandro R. P. da Rocha, Alexander Neuwelt, Paul B. Fisher, Devanand Sarkar, Xiang-Yang Wang, Joseph W. Landry, Rebecca K. Martin

**Affiliations:** 1 Massey Comprehensive Cancer Center, School of Medicine, Virginia Commonwealth University, Richmond, VA, United States; 2 Department of Cellular, Molecular and Genetic Medicine, School of Medicine, Virginia Commonwealth University, Richmond, VA, United States; 3 Department of Surgery, School of Medicine, Virginia Commonwealth University, Richmond, VA, United States; 4 Department of Pharmacology and Toxicology, School of Medicine, Virginia Commonwealth University, Richmond, VA, United States; 5 Department of Neurosurgery, School of Medicine, Virginia Commonwealth University, Richmond, VA, United States; 6 Department of Pharmaceutics, School of Pharmacy, Virginia Commonwealth University, Richmond, VA, United States; 7 Richmond Department of Veterans Affairs, Richmond, VA, United States; 8 Institute of Molecular Medicine, School of Medicine, Virginia Commonwealth University, Richmond, VA, United States; 9 Department of Microbiology and Immunology, School of Medicine, Virginia Commonwealth University, Richmond, VA, United States

**Keywords:** flow cytometry, immune phenotyping, mouse, antibodies, panel design and optimization

## Abstract

This 23-color mouse immunophenotyping panel was designed and developed by the Virginia Commonwealth University’s (VCU) flow cytometry shared resource (FCSR) to easily bring new use to our high-parameter spectral cytometers. Our method is broadly applicable to multiple tissue types, is modifiable, and provides a reproducible, cost-effective option for utilizing high-parameter flow cytometry. To facilitate the mouse immunophenotyping panel, researchers can be provided with optimized reagents, a step-by-step staining protocol, instrument training, pre-run single-color controls, and acquisition and analysis templates to streamline the workflow. Data analysis is generally done with a traditional manual gating strategy, but t-stochastic neighbor embedding (tSNE) and uniform manifold approximation projection (uMAP) generation can be performed, as desired. In an FCSR, this panel requires only light preparation work for shared resource (SR) staff with maximum benefit for researchers. Overall, this publication describes how SR facilities can provide additional benefits and services to their clientele by reducing costs, increasing reproducibility, and lowering the barriers of entry for researchers into the field of high parameter spectral flow cytometry. The panel described is used as an example of the application of the included methods, as well as a complete resource for other institutions to utilize themselves.

## 1 Introduction

### 1.1 Development of the protocol

With the advancement of spectral cytometry, high-dimensional flow analysis has become increasingly accessible for researchers. However, higher dimensionality introduces greater complexity in panel design, sample preparation, data acquisition, and analysis, so flow cytometry shared resources (FCSR) can play a critical role in bridging this gap for new users. The Virginia Commonwealth University’s (VCU) FCSR has developed a 23-color immunophenotyping panel (**Table 1**, **Supplementary Figure S1**) optimized for various mouse sample types, enabling comprehensive immune cell profiling at the single-cell level. Optimized multicolor immunofluorescence panels (OMIPs) are valuable resources for FCSR facilities to offer, and this current panel was created in collaboration with investigators that utilize the VCU FCSR (1–4) based on their needs and is currently provided as an example of how other institutions may do the same to expand their services to offer both broad accessibility and deep utility.

**Table 1:** Optimized 23-color mouse immunophenotyping panel.

Marker	Fluorescent tag	Laser	RRID	General purpose
CD45	BUV395	355	BD Biosciences Cat564279, RRID: AB_2651134	Pan leukocytes
F4-80	BUV496	355	BD Biosciences Cat750644, RRID: AB_2874772	Macrophages
CD4	BUV737	355	BD Biosciences Cat612761, RRID: AB_2870092	Helper T cells
CD279 (PD-1)	BV421	405	BioLegend Cat109121, RRID: AB_2687080	Functional marker for T or natural killer (NK) cells
MHC Class II (I-A)	eFluor 450	405	Thermo Fisher Scientific Cat48-5321-82, RRID: AB_1272204	B cells, dendritic cells, macrophages
CD90.2 (THY1.2)	BV480	405	BD Biosciences Cat746840, RRID: AB_2744090	Pan T cells
CD127 (IL-7Rα)	BV510	405	BD Biosciences Cat563353, RRID: AB_2738153	Innate lymphoid cells, exhausted T cells
CD44	BV570	405	BioLegend Cat103037, RRID: AB_10900641	Effector and memory T cells, activation
Ly-6G	BV605	405	BioLegend Cat127639, RRID: AB_2565880	Neutrophils, monocytes, myeloid-derived suppressor cells
CD19	BV650	405	BioLegend Cat115541, RRID: AB_11204087	Pan B cells
CD11c	BV711	405	BioLegend Cat117349, RRID: AB_2563905	Dendritic cells
TCRγδ	BV786	405	BD Biosciences Cat744117, RRID: AB_2742007	Pan γδ T cells
CD206	PerCP-eFluor 710	488	Thermo Fisher Scientific Cat46-2061-82, RRID: AB_2784688	M2 macrophages
FOXP3	Alexa Fluor 488	488	BioLegend Cat320012, RRID: AB_439748	Regulatory T cells
Ly-6C	PE	561	BioLegend Cat128008, RRID: AB_1186132	Monocytes, myeloid-derived suppressor cells
CD49b	PE-Dazzle 594	561	BioLegend Cat108924, RRID: AB_2565271	Natural killer (NK) cells
CD62L (L-Selectin)	PE-Cy5	561	BioLegend Cat104410, RRID: AB_313097	Effector and memory T cells
CD11b	PE-Fire 640	561	BioLegend Cat101280, RRID: AB_2888802	Myeloid cells
CD335 (NKp46)	PE-Cy7	561	Thermo Fisher Scientific Cat25-3351-82, RRID: AB_2573442	Natural killer (NK) cells
KLRG1	APC	640	BioLegend Cat138412, RRID: AB_10641560	Functional marker for T or natural killer (NK) cells
TCRβ	Alexa Fluor 700	640	BioLegend Cat109224, RRID: AB_1027648	Pan β T cells, natural killer T (NKT) cells
CD8	APC-Fire 750	640	BioLegend Cat100765, RRID: AB_2572112	Cytotoxic T cells
Zombie	NIR	640	No RRID available. BioLegend Cat423106	Viability

This panel includes a viability dye, 21 surface markers and 1 intracellular marker (FoxP3).

**Figure 1:**
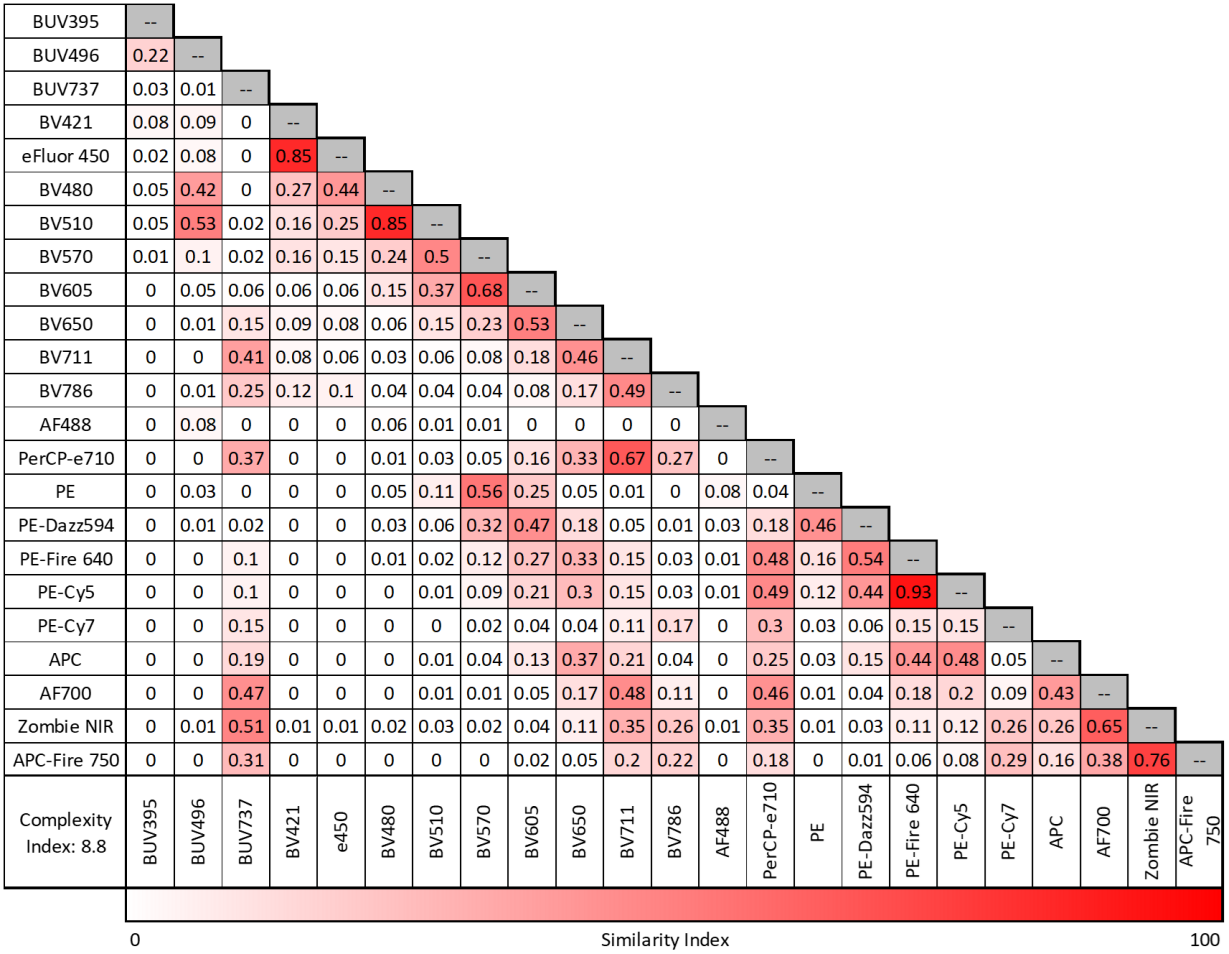
Similarity matrix and complexity index. Similarity matrix values closer to 1 indicate a high similarity between the two fluorophores. Complexity Index is a measure of how distinguishable a collection of spectral signatures are from each other and generally the lower the number, the less complex.

Designing high-parameter panels presents substantial challenges for investigators, particularly in matching antigen density levels and fluorophore brightness while minimizing spectral overlap. VCU’s FCSR mitigates these challenges by rigorously optimizing fluorophore selection and panel layout. **Figure 1** shows the similarity matrix and complexity index of the fluorophores in the panel from stained mouse splenocytes. The complexity index, also known as the condition number, is a measure of how distinguishable each of the spectral signatures is from each other. This is calculated by looking at the ratio of the largest over the least overlapping spectral signatures (5). Similarity values approaching 1 reflect highly overlapping spectra, which can result in increased spreading error and reduced resolution and can be particularly problematic for co-expressed markers (5). Higher spreading between co-expressed markers reduces the resolution between single and double positive populations. In **Supplementary Figure S2A**, the predicted loss of stain index quantifies the loss of separation between the positive and negative signal of a stain (column) due to the addition of a fluorophore (row), with higher values indicating that there is a greater loss in resolution. While spread can be impacted by the combination of fluorophores, the unmixing algorithm itself also introduces spread into the panel (5). **Supplementary Figure S2B** is a way to visualize which fluorophores have the greatest contribution to spreading through the unmixing algorithm. This figure shows the region of emission that should be avoided when adding additional fluorophores to reduce the amount of spreading when expanding the panel design (5, 6). For both matrices the higher values indicate a greater loss of resolution, so these markers are ideally kept on mutually exclusive populations.

### 1.2 Applications of the method

While current commercial panels, such as the Cytek^®^ 24-Color Mouse Immunoprofiling Panel, are available, these may not meet the specific needs of all institutions and often require a significant upfront investment from individual laboratories (Cytek, SKU R7-40014). To address these challenges, first, the described panel can be easily modified for each researcher’s needs, and secondly, having a centrally managed antibody bank, through a Shared Resource (SR) or laboratory, allows for bulk purchasing, optimal aliquoting, and cost-effective access to validated reagents. This method further reduces waste and avoids the need for investigators to individually purchase large volumes of antibodies to optimize high-parameter panels. The application of the described protocol provides an accessible entry point for less experienced researchers, while streamlining workflows for those with more experience. To ensure analytical consistency and reproducibility, standardized data acquisition and analysis templates can be created and provided to researchers to support best practices from start to finish. The entire process is designed with the intent to ease new clients of an SR into performing higher parameter flow cytometry.

The 5-laser Cytek Aurora spectral flow cytometer allows for up to 64 parameters to be run simultaneously, and the described panel offers sufficient flexibility for customizations for each researcher’s goal. The optical arrangement for the panel, on a 5-laser Cytek Aurora instrument, shows which laser each of the 23 fluorophores is excited by as well as their maximum emission (**Supplementary Figure S3**). Fluorophores are selected to minimize having too many within a similar emission wavelength, which can contribute to additional spreading. One customizable option involves removing the intracellular FoxP3 marker and replacing it with another Alexa Fluor 488 (AF488) conjugated antibody. This substitution is favorable, as AF488 is readily available for most commercial markers and the other fluorophores in the panel have low similarity to AF488, as shown in **Figure 1**, meaning the additional marker can be co-expressed with the original markers without causing a loss of resolution or spreading. Alternatively, users have a variety of options for adding new fluorophores into the existing panel. **Table 2** lists some additional fluorophores that may be incorporated without significantly increasing the panel’s complexity index and notes where additional spreading is most likely to occur.

**Table 2:** Potential additional fluorophore options for the described panel.

Additional fluorophore	Adjusted complexity index	Delta complexity	Potential spread with
BUV615	8.95	+0.15	PE-Dazzle594
BUV563	8.95	+0.15	PE
BUV661	9.35	+0.55	APC
BUV805	8.81	+0.01	
BB630	9.04	+0.24	BV605
PerCP-Cy5.5	14.6	+5.8	PerCP-efluor710 and BV711
BB755	9.29	+0.49	BV786
BB790	8.98	+0.18	BV786
cFluor BYG750	9.06	+0.26	
PE-Fire 810	8.85	+0.05	PE
cFluor R685	9.02	+0.22	PE-Cy5 and AF700

The delta complexity is based off the original complexity index of 8.8.

**Figure 2:**
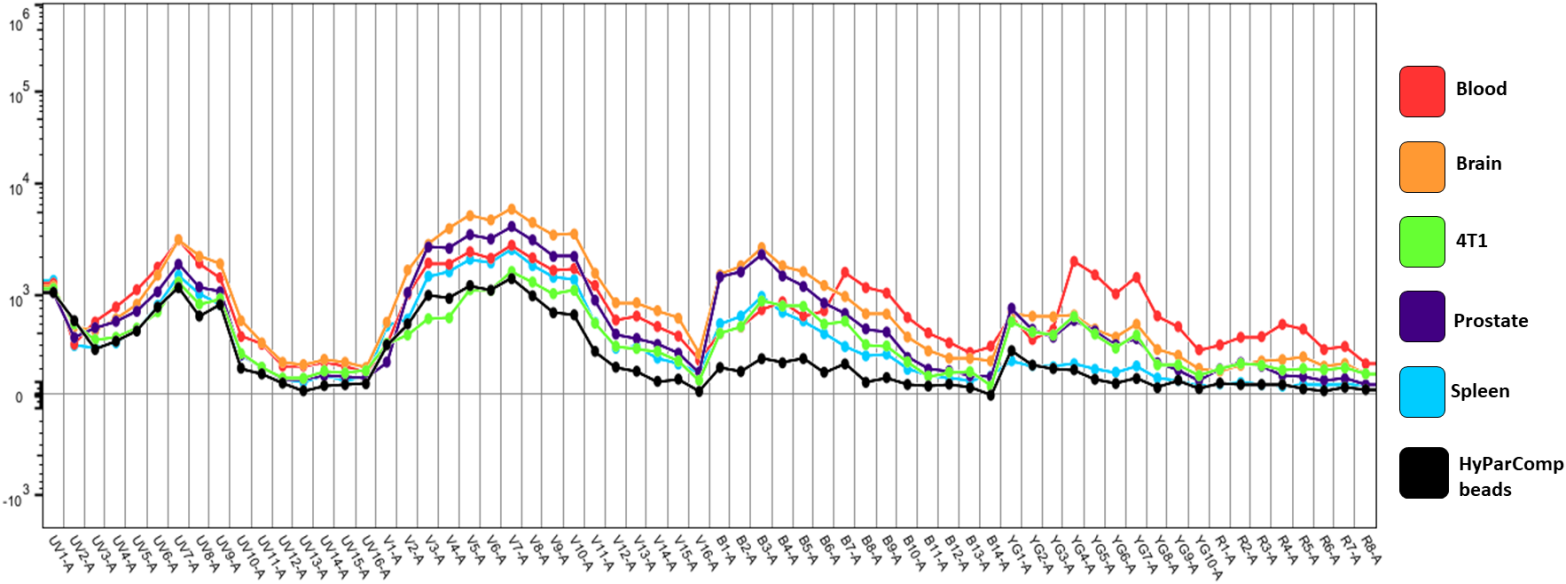
Auto fluorescence of reference groups and different sample types. HyParComp beads and unstained naïve splenocytes are used for the reference group during unmixing. Other sample types include prostate tumor cells, breast tumor cells (4T1), brain cells and blood.

To further enhance resolution, the Cytek Aurora system supports autofluorescence extraction, which allows for improved resolution of stained populations. While VCU’s FCSR does provide reference controls, variability in tissue-specific autofluorescence can impact signal quality. Since fluorescent signals are additive, having the proper background to subtract from the true fluorescent signal is important, so researchers are encouraged to prepare an unstained control per sample type (see Note 1). As illustrated in **Figure 2**, the auto fluorescence signatures, generated in Flowjo Spectral Population Viewer (BD Biosciences), in naïve spleen samples and HyParComp cell mimics (HyParComp beads, Slingshot Biosciences) are lower than that observed in blood or enzymatically digested tissue. Including appropriate controls allows for accurate subtraction of autofluorescence and an improved signal-to-noise ratio.

**Figure 3:**
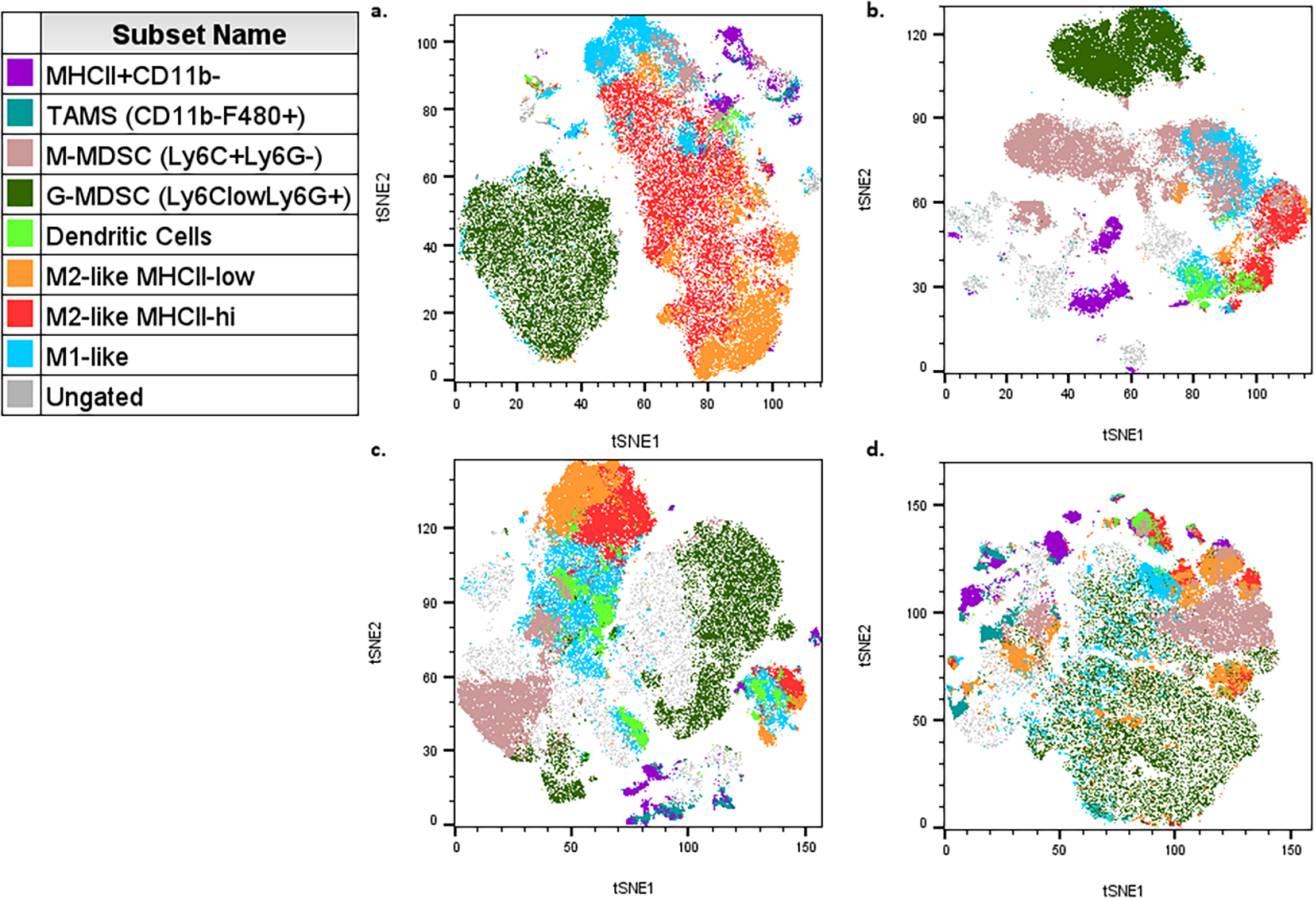
Example tSNE of myeloid populations in different tumor microenvironments (TMEs). **(a)** 4T1 cells injected SQ into mouse flank then taken 13 days after injection (n=5). **(b)** HiMYC spontaneously developing prostate tumor (n=5). **(c)** LLC cells surgically implanted into mouse flank then taken 2 weeks later (n=3). **(d)** Prostate cancer cells injected into the tibia of mice and then taken 7 days after injection (n=5).

**Figure 4:**
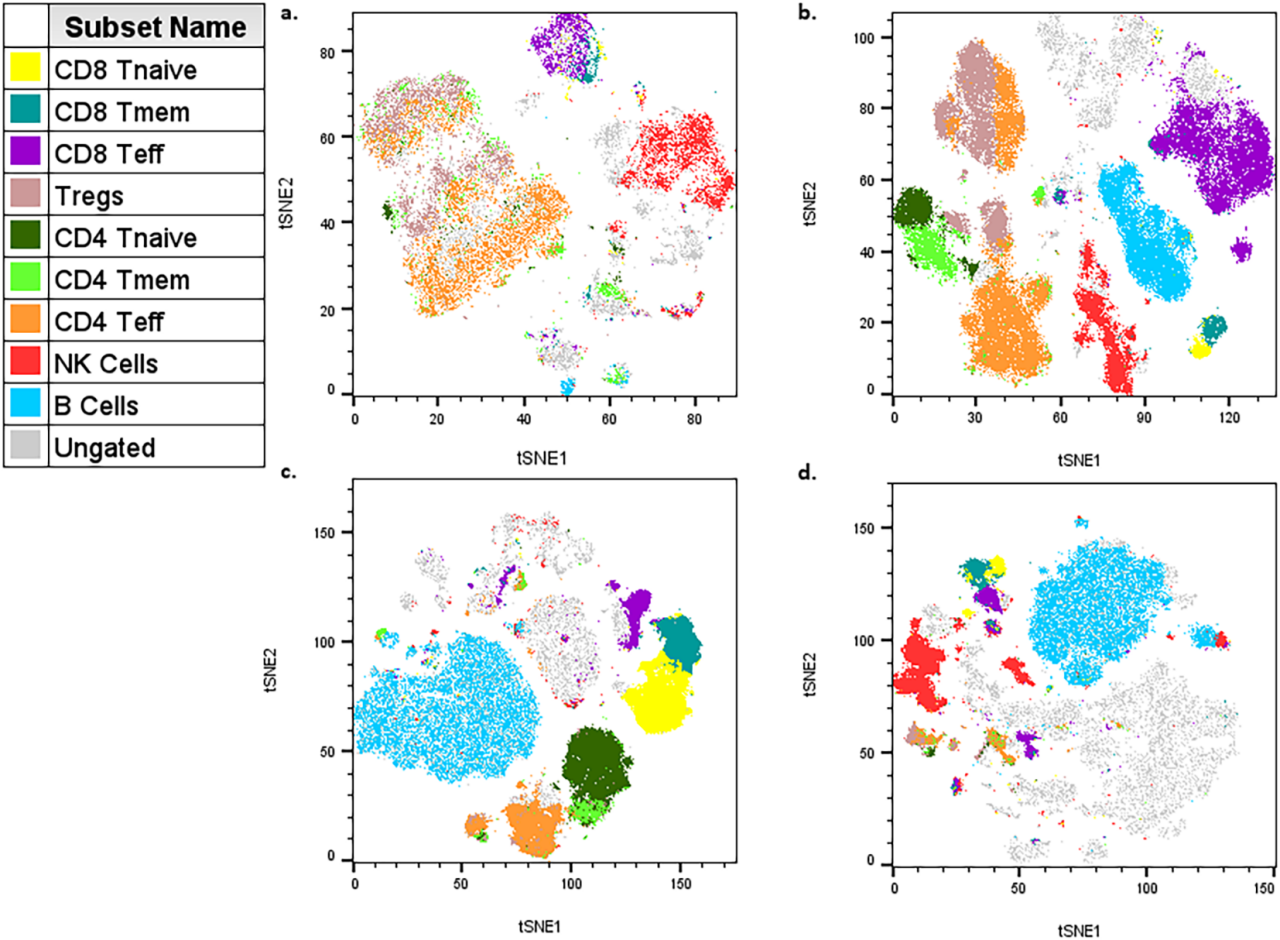
Example tSNE of lymphoid populations in different tumor microenvironments (TMEs). **(a)** 4T1 cells injected SQ into mouse flank then taken 13 days after injection (n=5). **(b)** HiMYC spontaneously developing prostate tumor (n=5). **(c)** LLC cells surgically implanted into mouse flank then taken 2 weeks later (n=3). **(d)** Prostate cancer cells injected into the tibia of mice and then taken 7 days after injection (n=5).

**Figure 5:**
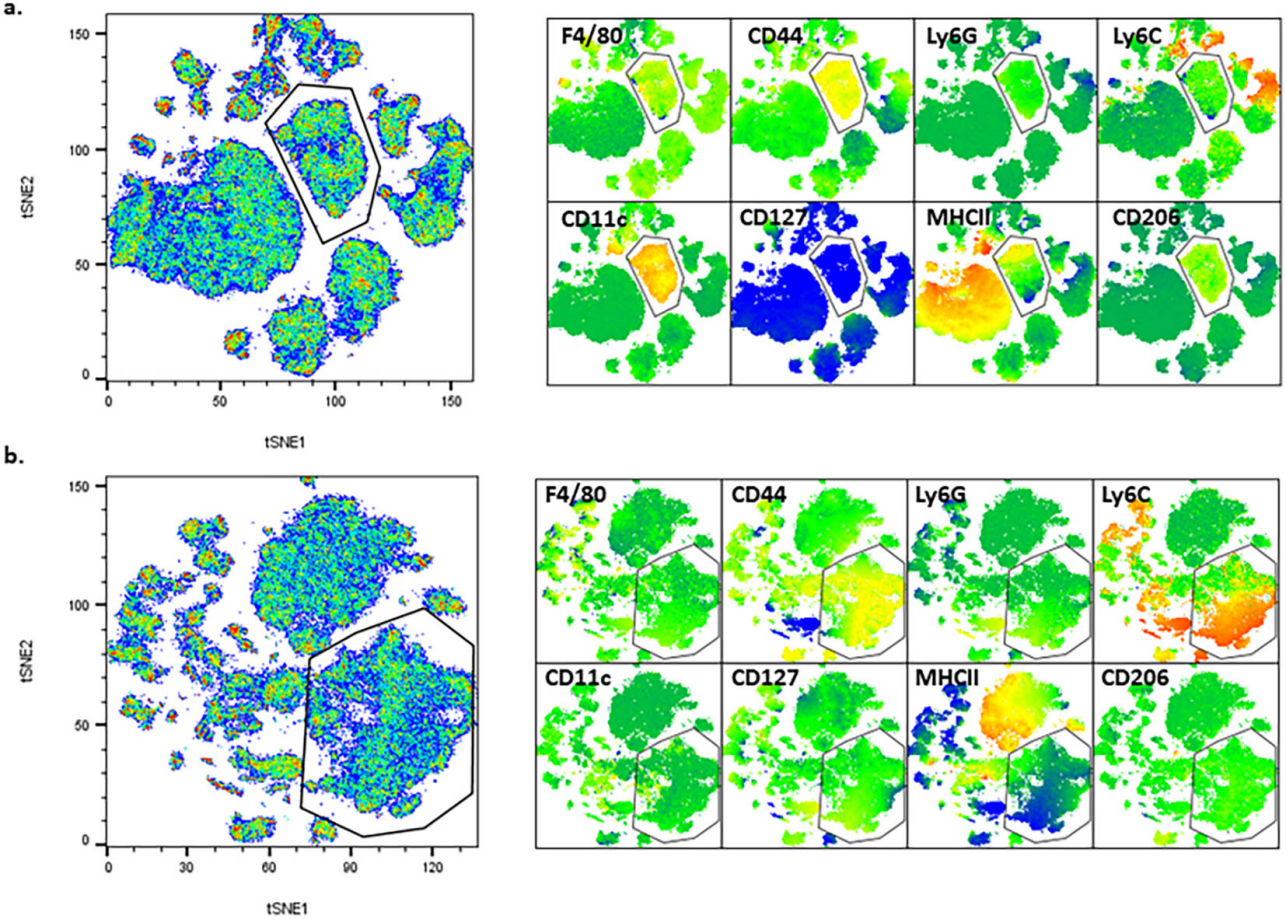
Heatmaps of lymphoid tSNEs in different tumor microenvironments (TMEs). **(a)** LLC cells surgically implanted into mouse flank then taken 2 weeks later (n=3). **(b)** Prostate cancer cells injected into the tibia of mice and then taken 7 days after injection (n=5). Multigraph heat map overlays applied for additional analysis of ungated populations.

For data analysis, the described panel supports both conventional gating and high-dimensional analysis, such as t-stochastic neighbor embedding (tSNE) and uniform manifold approximation projection (uMAP) figure generation. High parameter analysis is particularly powerful in studying heterogeneous microenvironments, such as the tumor microenvironment (TME) (1, 2, 4, 7, 8). Manual gating was performed, and populations were overlaid on each tSNE for figures. Each tumor type exhibits distinct immune profiles, and this panel allows for characterization of different populations from both myeloid (**Figure 3**, **Supplementary Figure S4**) and lymphoid (**Figure 4**, **Supplementary Figure S5**) lineages for multiple tumor models (see Note 15) (7–10). Even with well-defined manual gates, unsupervised clustering algorithms can still be useful for identifying novel populations (FlowJo, LLC). The myeloid tSNE populations (**Figure 3**) were gated for viable CD45+ CD19- and TCRβ- TCRγδ- to exclude any B and T cell populations, respectively, while the lymphoid tSNE populations (**Figure 4**) were gated for viable CD45+ CD11b- cells to exclude most myeloid cells. Although CD11b can be used as a pan-myeloid marker, there are still other CD11b-myeloid populations (8, 11). For instance, in **Figures 4C, D**, there are some ungated (gray) islands in the TME, but upon further analysis utilizing the multigraph overlay function (**Figure 5**), we are able to see these islands are positive for other populational markers in the panel, that could be overlooked by manual gating bias. For myeloid populations, typically gating is done on CD11b+ cells prior to any downstream analysis, but as shown in **Figure 5**, if gating is done on the positive population first, these other myeloid populations that are CD11b- might be missed (8, 11). In **Figure 5A**, there is an ungated population of dendritic cells (outlined) that are CD11b-CD11c+CD44+, and in **Figure 5B** there is an ungated population (outlined) of CD11b-Ly6C+CD44+ myeloid cells. This demonstrates how manual gating can be biased, especially for those less familiar with immune cell markers. This also reinforces the concept that the “recommended” gating strategy and analysis templates are just a general example and should be modified for each researcher’s goals. To help reduce bias, dimensionality reduction techniques, such as tSNE plot visualization, can be a more useful tool for seeing all the selected parameters and samples at the same time to help identify rare cell types (12). Understanding and being able to modulate the TME is a major goal for cancer researchers, and this optimized panel allows for researchers who are new to flow to jump right into high-parameter flow analysis of immune cells to benefit their research goals.

**Figure 6:**
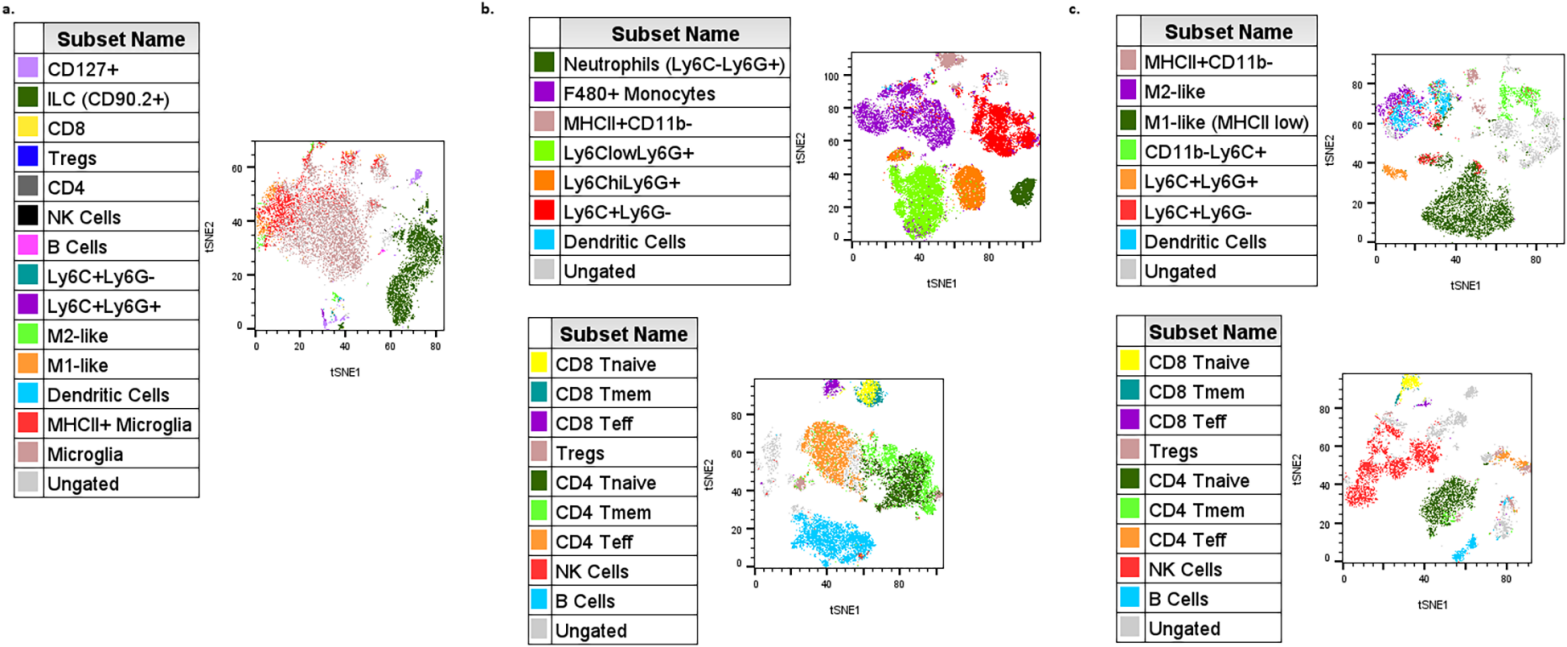
Example tSNEs of myeloid and lymphoid populations in various sample types. **(a)** Populations in the brain (n=3). **(b)** Populations in blood (n=5). **(c)** Populations in mammary gland (n=4).

**Figure 7:**
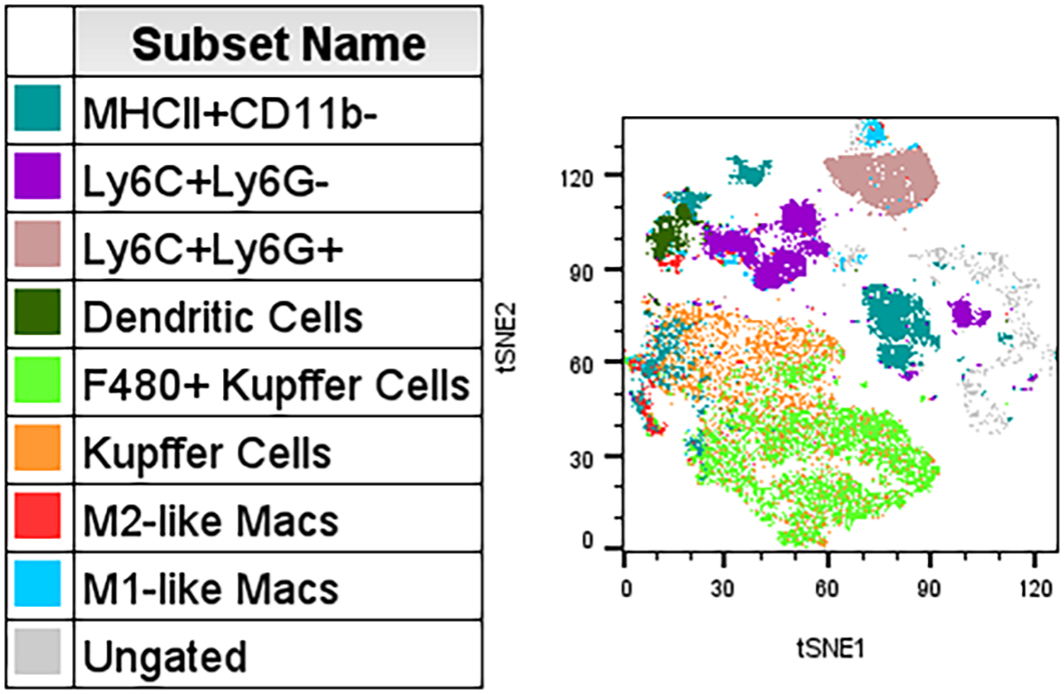
Example tSNE of myeloid populations in the liver with customizations to described panel. Myeloid populations with substitutions (replace APC - KLRG1 for APC - TIM4) and additions (PerCPCy5.5 – Clec2) to the panel. Kupffer cells were gated on CD11b+ then TIM4+Clec2+/- cells were gated on expression of F4/80. M1 and M2 macrophages were gated on TIM4-Clec2- cells prior to MHCII and CD206 expression. (n=3).

Beyond tumor studies, this panel is applicable across blood and various tissue types. Using this panel in the brain enables identification of microglia as well as infiltrating immune cells like macrophages and innate lymphoid cells (ILCs) (**Figure 6A**, **Supplementary Figures S6A**, **S7**). In the blood, users can differentiation monocyte subsets as well as the activation states of T cells (**Figure 6B**, **Supplementary Figure S6B**). This panel is also effective in other digested tissues like mammary glands (**Figure 6C**, **Supplementary Figure S6C**). Overall, this panel is aimed more directly at researchers performing exploratory immune cell analysis, which allows for future directions to focus on more specific populations after identifying general trends. Liver resident macrophages, or Kupffer cells, are of interest to many investigators, and substitutions (replace APC - KLRG1 with APC - TIM4) and additions (PerCPCy5.5 - Clec2) to this panel can allow for differentiation of Kupffer cells from infiltrating macrophages (**Figure 7**, **Supplementary Figure S8**) (13). Collectively, these examples underscore the panel’s versatility across tissues, including those requiring complex dissociation steps. Although gated populations may vary based on the experimental context, **Figures 3**-**7** offer general examples of how this panel can be applied to characterize diverse immune subsets in exploratory studies (see Note 15).

## 2 Materials

### 2.1 Equipment

Cytek Aurora analyzer with five lasers (355, 405, 488, 561, 640 nm) and automated 96-well plate loader (optional) (see Note 2). An acquisition template is saved on the instrument software, which includes all reference group single color controls, as well as the instrument Forward Scatter/FSC-A and Side Scatter/SSC-A detectors set up for lymphocytes, changes to instrument settings are made as needed and can be saved on the instrument software for future use. Acquisition templates and FSC-A/SSC-A settings are instrument-specific and should be modified accordingly.

### 2.2 Reagents

- Zombie NIR – This is a viability dye to allow for dead cell exclusion during the analysis
- FACS buffer (1× PBS with 2% bovine serum albumin or 2% fetal bovine serum)
- Fc blocking mix: purified clone 2.4G2 made in house from hybridoma (14), or preferred commercial vendor, FACS buffer
- Master Antibody Mix: antibody cocktail of the 21 cell surface markers, Brilliant Stain Buffer (BD Biosciences Cat# 563794), FACS buffer
- Fixation and Permeabilization Buffer (BioLegend Cat# 424401 or BD Biosciences Cat# 562574)
- FoxP3 Intracellular Mix: FoxP3 antibody, FACS buffer

### 2.3 Step-by-step staining procedure

1. Antibody cocktails can be made prior to staining. Fixation and permeabilization buffers should be diluted fresh (see Note 3). After preparation, make single-cell suspensions of samples (see Note 4).
2. Count cells with trypan blue or a similar cell staining dye—start with 1e^6^ cells per sample, if there is less than that, then use everything (see Note 5).
3. OPTIONAL: This step is for additional “count” analysis—most flow analysis is shown as a percentage of a population; however, the Cytek Aurora measures the exact volume run from each sample so calculations can be done to get the total cell count of the organ weight/blood volume/total sample, and so forth (see Note 6).
4. Staining may be performed in either 96-well U-bottom plates or in regular 5 ml flow tubes. This staining protocol contains volumes for both methods (see Note 7).
5. CRITICAL: All samples and reagents should be kept on ice for the entire protocol, unless otherwise stated. After making a single-cell suspension, counting cells, and adding them to the plate. CRITICAL: wash samples with 100 µl of 1× PBS. Spin at 350 g for 5 min at 4°C and decant.
6. Add Zombie NIR stain master mix –100 µl per sample (see Note 8). Mix well (see Note 9). Incubate in the dark at room temperature for 10 min (see Note 10).
7. To stop the reaction, add 100 µl FACS buffer to the samples. Spin and decant as in Step 5 above (see Note 11).
8. Resuspend cells in 50 µl FACS buffer. Add 10 µl Fc Blocking Mix per sample, mix well. Let incubate at 4°C for 5–10 min. CRITICAL: Do not wash out the Fc Blocking Mix following the incubation.
9. Add 50 µl Stain Master Mix per sample, mix well. Incubate at 4°C in the dark for 30 min.
10. Wash with 100 µl FACS buffer. Spin and decant as in Step 5 above.
11. Fixation Step: Use the True Nuclear Transcription Factor Fixation Buffer and fix according to manufacturer’s protocol. Of note, BioLegend and BD Biosciences have slightly different protocols.
12. OPTIONAL: If the protocol does not require FoxP3 staining –
  a. Option 1 – No fixation. After Step 10, resuspend cells in at least 100 µl of FACS buffer and immediately acquire samples.
  b. Option 2 – Fixation with True Nuclear Transcription Factor Buffer. After following the fixation protocol in Step 11, resuspend in at least 100 µl FACS buffer and store at 4°C in the dark until ready to acquire samples (fixed samples can be stored for up to 5 days).
  c. Option 3 – Paraformaldehyde fixation. After Step 10, resuspend cells in 2%–4% paraformaldehyde (PFA) and leave at room temperature for 15 min for fixation. Wash with 100 µl FACS buffer. Spin and decant as above. Resuspend in at least 100 µl FACS buffer and store at 4°C in the dark until ready to acquire samples (fixed samples can be stored for up to 5 days).
13. Permeabilization Step - Perform per manufacturer’s protocol. Of note, BioLegend and BD Biosciences have slightly different protocols. CRITICAL: After permeabilizing the cells all washes moving forward should be done with the permeabilization buffer—do NOT use FACS buffer.
14. After washes with the permeabilization buffer, perform the intracellular FoxP3 staining. Add 100 µl permeabilization buffer to each sample, mix well. Add 10 µl Fc Blocking Mix, incubate for 5 min at 4°C. CRITICAL: Do not wash out. Then add 10 µl FoxP3 Stain Mix per sample.
  a. PAUSE POINT: FoxP3 may be stained from 4h to overnight at 4°C in the dark (15). The manufacture’s protocol recommends 40 min–1h at 4 °C in the dark.
15. After incubation with FoxP3, wash with permeabilization buffer. Spin and decant as above.
16. Repeat this wash step.
17. Re-suspend samples in at least 100 µl of FACS buffer (see Note 12). Store samples at 4°C, covered from light (fixed samples can be stored for up to 5 days).

#### 2.3.1 Timing of procedure

Step 1–5: *This is dependent on each researcher’s experiment.* If doing blood collection this could take up to 1h, depending on number of mice used. If doing digestion of brain or other tissue this could take more than 2h. Researchers are encouraged to keep samples on ice during sample collection.

Step 6–11: If doing the recommended fixation with the True Nuclear Transcription Factor Fixation Buffer, *the completion of these steps may take roughly 1.5–2h.*

Step 12–13: If doing a shorter incubation time, per manufacture’s protocol, *the completion of these steps may take roughly 1.5h*. If doing the recommended overnight incubation of intracellular FoxP3, *the completion of these steps may take roughly 20 min, followed by the overnight incubation at 4°C.*

Step 14–16: Regardless of intracellular incubation, *the completion of these steps may take roughly 15 minutes.* For troubleshooting help, please refer to **Table 3**.

**Table 3:** Troubleshooting table.

Step	Problem	Possible Reason	Solution
7	No pellet	Cell count was wrong	Confirm cell count protocol
		Lysis of red blood cells (RBCs) was too long, and all cells were lysed	Confirm RBC lysis protocol is correct
Data Analysis	Cells are all Zombie NIR positive	Cells were dead prior to Zombie staining	Optimize digestion protocol and/or timing of sample collection
	Cells are all Zombie NIR negative	Zombie staining was done in media with protein	Staining and resuspension of Zombie needs to be done in protein-free media like 1X PBS
	Antibody signals are dim	Old antibody cocktails were used	Mixed antibody cocktails are stable for up to 10 days
		Too many cells were used for staining	Count cells prior to staining to ensure ~ 1e6 cells per sample
	Can’t see certain subpopulations in tSNE/uMAP	One cell population makes up the majority of events	Perform density-dependent down sampling on the population prior to generating tSNE/uMAP. Density dependent down sampling can be performed in FCS Express 7 or FlowJo using the Downsample plugin (*see* Note 16), and R using the tidytof package^18^. Density dependent down sampling can emphasize smaller/rarer populations while reducing the impact of larger populations for the purpose of visualization, utilizing SPADE algorithm (16).

### 2.4 Data analysis and gating strategy

The described panel broadly identifies myeloid and lymphoid immune cell populations including: dendritic cells (DCs), neutrophils, macrophages, monocytes, CD4 and CD8 T cells, regulatory T cells, T γδ cells, natural killer (NK)/NK T cells, ILCs, and B cells. The panel also includes markers for effector and memory cells as well as functional markers. A fluorescence minus one (FMO) control is suggested for FoxP3 to use as a negative gating control for the T-regulatory cells (see Note 13).

**Figure 8:**
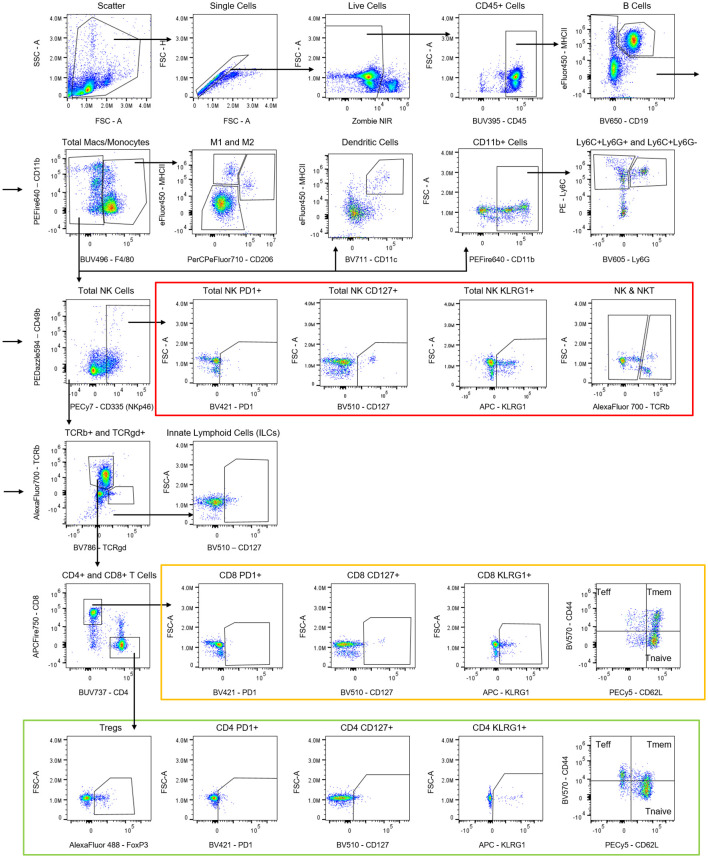
Suggested gating strategy for the 23-color immunophenotyping panel.

Initial leukocyte gating is performed on scatter and viability to remove aggregates, doublets, and dead cells. From this cleaned up population, leukocytes are gated based on CD45+ expression (**Figure 8**).

#### 2.4.1 Myeloid subsets

CD45+ cells are then gated for the myeloid populations, broadly defined as CD11b+. Macrophages are further defined by F4/80+ expression and can then be delineate into M1-like (CD206−) and M2-like (CD206+) as well as MHCII+ macrophages. Dendritic cells are defined by CD11c+MHCII+F4/80− expression. Neutrophils can be defined as F480-Ly6C-Ly6G+ cells and other monocytes can be separated based on Ly6C and Ly6G expression. In tumor samples, myeloid derived suppressor cells can be delineated into the monocytic (Ly6C+Ly6G−) and granulocytic (Ly6C+Ly6G+) subsets (**Figure 8**). Myeloid subpopulation gating can become very complex and nuanced, as many of the markers are expressed on multiple cell types (see Note 14).

#### 2.4.2 Lymphocyte subsets

Lymphocytes are gated on F4/80− cells, due to their frequency in non-specifically binding to antibodies via their high uptake of antibody and high expression of Fc-receptors (17). After gating on F4/80− cells, the cells are separated by CD19+MHCII+ expression to identify B cells. The CD19-MHCII− population is then gated on either TCRβ+ or TCRγδ+ T cells. The TCRβ population is separated into CD4 and CD8 T cells, which are both then further separated into effector (CD44+CD62L−), memory (CD44+CD62L+), and naïve (CD44-CD62L+) T-cell populations. This panel does not include CD3, due to its internalization rate, and instead uses TCRβ and/or CD90.2 to define the T cell populations (18, 19). It should be noted that although CD90.2 is present on most common mouse strains (Balb/c, CBA/J, C3H/He, C57BL/−, DBA, NZB/−), it is not present on certain mice strains (AKR, BDP, MA/MyJ). The TCRβ clone (H57-597) is present on all tested mice strains. Overall CD90 and/or CD90.1 could be substituted into the panel if needed. Both CD8 and CD4 T cells are also analyzed for the expression of PD1, CD127, and KLRG1. The transcription factor FoxP3 is optional in the panel to define CD4+ T-regulatory cells (**Figure 8**).

#### 2.4.3 Natural killer cells and innate lymphoid cells

In parallel to the gating of the T cells, off of the CD19-MHCII-population, total NK cells are identified by CD335+CD49b+/−. NK T cells are identified based on expression of TCRβ. Similar to T cells, total NK cells are examined for expression of PD1, CD127 and KLRG1 (**Figure 8**).

After CD45+ gating, ILCs are negative for lineage markers (TCRβ/CD19/CD11c/CD11b) and positive for CD127 (**Figure 8**).

### 2.5 Notes

1. Unstained and viability dye only controls are needed for unmixing with autofluorescence extraction and for analysis, respectively. Viability only controls are also helpful to confirm consistent viability between runs.
2. This panel could potentially be run on a 4 laser Cytek Aurora analyzer (no UV laser). BUV496 and BUV737 are partially excited by a violet laser and can be analyzed from those emission channels. BUV395 would be left out of the panel, since BUV395 is on CD45 this marker is overall used to get rid of other non-leukocyte cells, but the other cell populations can still be defined without it.
3. Ideally, reagents may be prepared up to 24 hours prior to sample collection and staining. The Master Antibody Mix contains Brilliant Stain Buffer to prevent polymerization of antibodies and is prepared at a concentration ideal for 1e^6^ cells, based on splenocytes. Stock and recommended working concentrations are listed in **Supplementary Table S1** for viability dye, Fc block, surface, and intracellular antibodies. Further Zombie NIR information can be found in Note 8. Fc block should be used at the recommended concentration, and FACS buffer added to bring the final volume to 10 µl per sample (of note, Fc blocking is done before surface staining and before intracellular FoxP3 staining). The 21 surface antibodies can be added to the Master Antibody Mix at the recommended concentration (**Supplementary Table S1**), Brilliant Staining Buffer can be added at 7 µl per sample (this has been titrated from the manufacturer’s recommendation), FACS buffer is then added to bring to final volume up to 50 µl per sample. Intracellular FoxP3 Mix can be made by adding the recommended concentration, and FACS buffer can be used to bring the total volume up to 10 µl per sample. Fc Blocking Mix, Master Antibody Mix and Intracellular FoxP3 Mix can be stored covered from light at 4°C for up to 10 days. Fresh single-color reference controls are run routinely on both mouse splenocytes and HyParComp beads (Slingshot Biosciences Cat# SSB-05-A). FACS Buffer can be made in advance and stored at 4°C. Fixation and Permeabilization Buffers should be stored per manufacturers protocol and dilutions should be done fresh, the day of sample collection and staining.
4. Tailored organ processing details are not included in this protocol.
5. Optional: This is a useful step to gauge viability of the cells prior to starting the staining protocol but if the sample is known to be less than 1e6 cells per sample then skip this step and use the full sample available.
6. Optional “Count” analysis: Researchers will need to make note of three things: (1) How much organ/tissue/blood volume was initially obtained. (2) How much of that is being used for flow. (3) What volume was used to resuspend cells in at the very end of the staining protocol. This will allow the count analysis to be extrapolated back to either whole organ count or count per weight or total volume and so forth.
*Please read through the four examples below before starting the experimental takedown*
  i. If processing an entire spleen to digest - then take one-third of the volume of the spleen digestion to stain for flow. The final resuspension is 300 µl to run on the instrument.
  ii. If bleeding a mouse, collect 200 µl of blood in heparin. Then prepare that blood for flow and stain the entire sample. Re-suspend the final sample in 300 µl to run on the instrument.
  iii. Lavage each mouse in the experiment with the same volume. Record the recovered volume of lavage fluid. Stain half of this recovered sample, then re-suspend this sample in 200 µl to run on the instrument.
  iv. If collecting a tumor—weigh the tumor, then cut off a piece for flow and weigh the piece for flow. Digest the piece for flow and stain everything for flow. Re-suspend the sample in 250 µl to run on the instrument.
7. When working with tube volumes, Adding Fc Blocking Mix, Master Stain Mix, and FoxP3 Mix are the same volumes as used in plate staining. Washing should be done with 1 ml of FACS or Permeabilization buffer. Fixation should be done in 1 ml of fixation buffer.
8. How to prepare Zombie NIR: Zombie NIR is re-suspended per manufacturer’s instructions upon arrival in 100 µl DMSO (can be aliquoted into smaller volumes and stored like this at −20°C). Zombie NIR is an amine reactive dye and needs to be stained and resuspended in a protein free media (1× PBS) to avoid a false negative result.
  a. For each use, remove aliquot from freezer and thaw. Make a stain master mix with 0.5 µl Zombie NIR in 99.5 µL PBS per sample. CRITICAL: The manufacturer’s instructions call for 1 µl per sample, this has been titrated down to half this volume to account for excessive brightness and off-scale visualization on the spectral flow cytometer.
  b. Return Zombie NIR stock to −20°C.
9. Mixing with plates or tubes: For plates, to mix each well pipette up and down 3–5 times to ensure sample is resuspended. For tubes, vortex or pipette mix the tubes.
10. Include one unstained/negative sample per organ (cells may be pooled from different treatment groups for this control, it does not need to contain 1e^6^ cells)—make sure the experiment contains one well or tube with no zombie NIR staining and no antibody staining for proper unmixing and autofluorescence extraction.
11. Look at the bottom of the plate or tube—a pellet should be visible. Continue examining the bottom of the plate or tube throughout the protocol to ensure excessive cell loss is not occurring. After the fixation step it is normal for the pellet to become smaller and harder to see.
12. For the optional “count” analysis, make note of final resuspension volume
13. Researchers should add the Zombie NIR, Fc Blocking Mix and Master Antibody Mix to the FMO control well or tube and fix and permeabilize the cells the same way as the rest of the samples, but do not add the intracellular FoxP3 mix to this FMO control.
14. This is an example of just one strategy for gating, there are multiple different ways to do the gating, depending on each researcher’s interest and any substitutions/alterations to the antibody stains in the panel.
15. For the myeloid tSNE generation in FlowJo, control/vehicle samples were concatenated after gating on single, viable, CD45+, TCRβ-TCRγδ-CD19 cells. Myeloid tSNE’s were run with the following parameters: CD11b, CD11c, F4/80, Ly6C, Ly6G, MHCII, CD206, CD44, CD62L, CD49b, CD90.2, CD127, KLRG1, PD1, CD4, CD8. For the lymphoid tSNE generation in Flowjo, control/vehicles samples were concatenated after gating on single, viable, CD45+, CD11b- cells. Lymphoid tSNE’s were run with the following parameters: TCRβ, TCRγδ, CD4, CD8, FoxP3, CD49b, CD335 (NKp46), CD19, MHCII, CD44, CD62L, KLRG1, PD1, CD90.2, CD127. For both myeloid and lymphoid tSNEs, the Approximate (random projection forest - ANNOY) KNN algorithm was used with the FTT Interpolation (Flt-SNE) used for the gradient algorithm^11^. Iterations were left at the default value of 1,000, perplexity was increased from 30 to 200 and the learning rate was left at the default value, which varies per group. Populations from manual gating were applied to the tSNEs. Example data shown were taken from VCU’s FCSR user data with their explicit permission and only control samples were included for the analysis. These parameters can be adjusted for each researcher’s interest and only describe the samples visualized in this manuscript. Details are not provided on experimental preparation prior to staining with the 23-color panel since these are just examples of applicability of the panel.

## 3 Conclusions

The emergence of spectral cytometry has allowed for investigators to utilize flow cytometry as an essential tool in the ever-expanding field of high-parameter single-cell data analysis. The panel described by VCU’s FCSR exemplifies how SR labs can optimize custom immunophenotyping panels to save monetary resources for investigators while still getting cutting-edge data. The described panel also allows for researchers to acclimate to high-parameter cytometry and get hands-on experience in instrument operation and complex data analysis that they can utilize for development of more hypothesis-driven flow cytometry panels in a more controlled experimental environment.

## Data Availability

The raw data supporting the conclusions of this article will be made available by the authors, without undue reservation.
