# Supplementary material for: Making deep immunophenotyping accessible: the successful application of a guided 23-parameter mouse immunophenotyping panel package provided through a shared resource
Source: Front Immunol. 2026 Jan 9;16:1668405. doi: 10.3389/fimmu.2025.1668405 (PMC12827670; doi:10.3389/fimmu.2025.1668405)
Supplement: Supplementary file 1 [file Presentation1.pptx]

## Slide 1
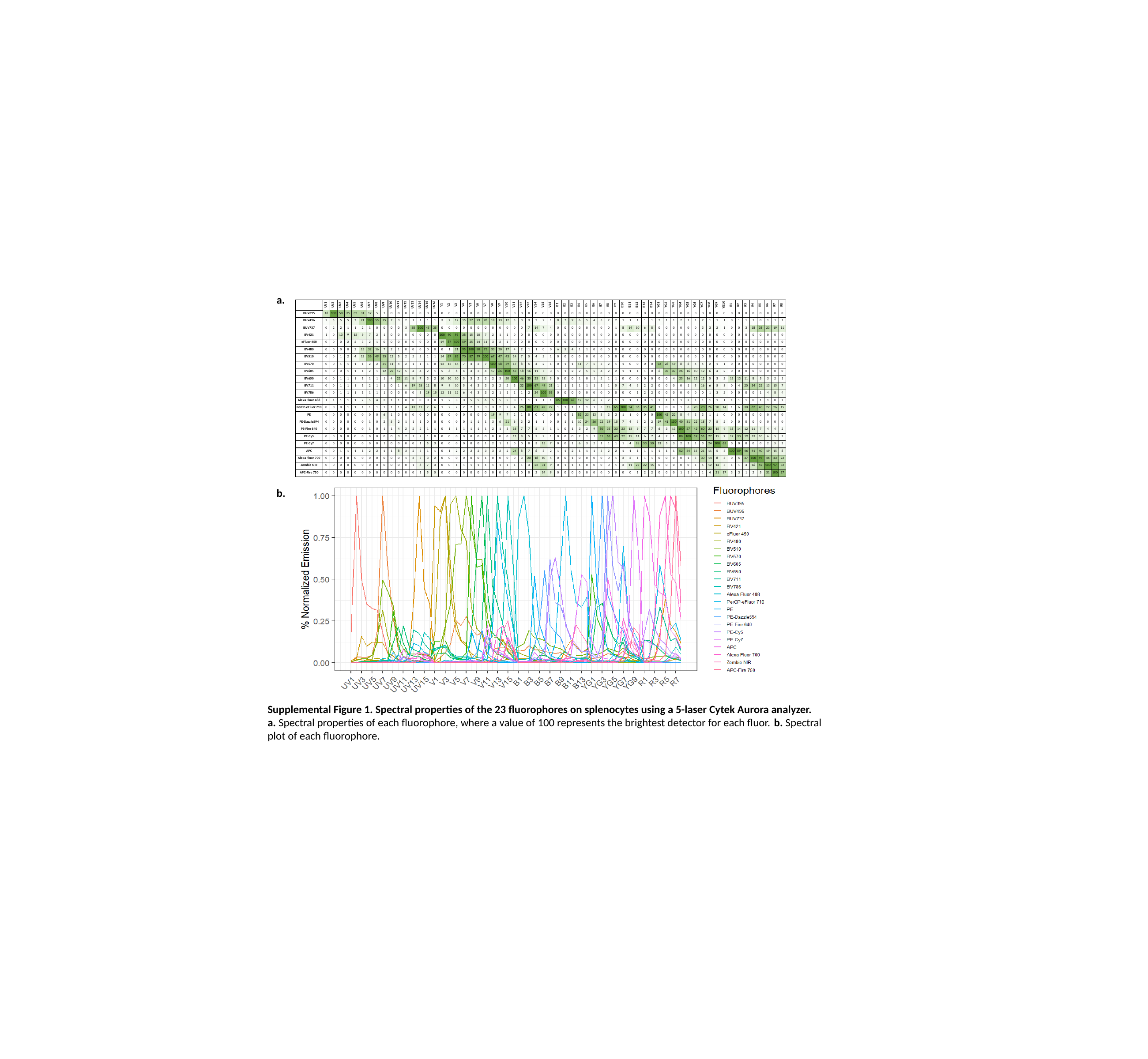

a.
b.
Supplemental Figure 1. Spectral properties of the 23 fluorophores on splenocytes using a 5-laser Cytek Aurora analyzer. a. Spectral properties of each fluorophore, where a value of 100 represents the brightest detector for each fluor. b. Spectral plot of each fluorophore.

## Slide 2
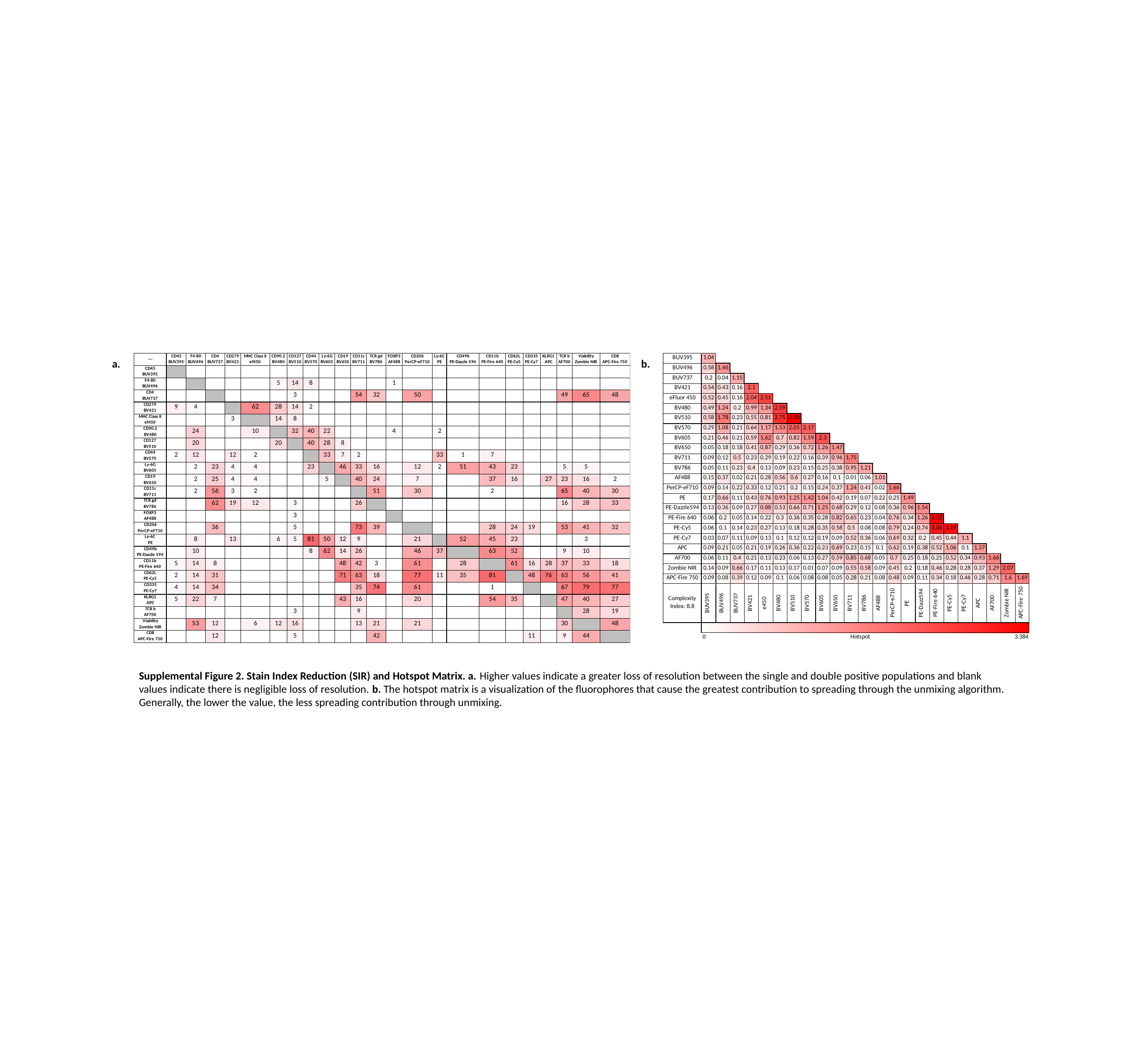

a.
b.
Supplemental Figure 2. Stain Index Reduction (SIR) and Hotspot Matrix. a. Higher values indicate a greater loss of resolution between the single and double positive populations and blank values indicate there is negligible loss of resolution. b. The hotspot matrix is a visualization of the fluorophores that cause the greatest contribution to spreading through the unmixing algorithm. Generally, the lower the value, the less spreading contribution through unmixing.

## Slide 3
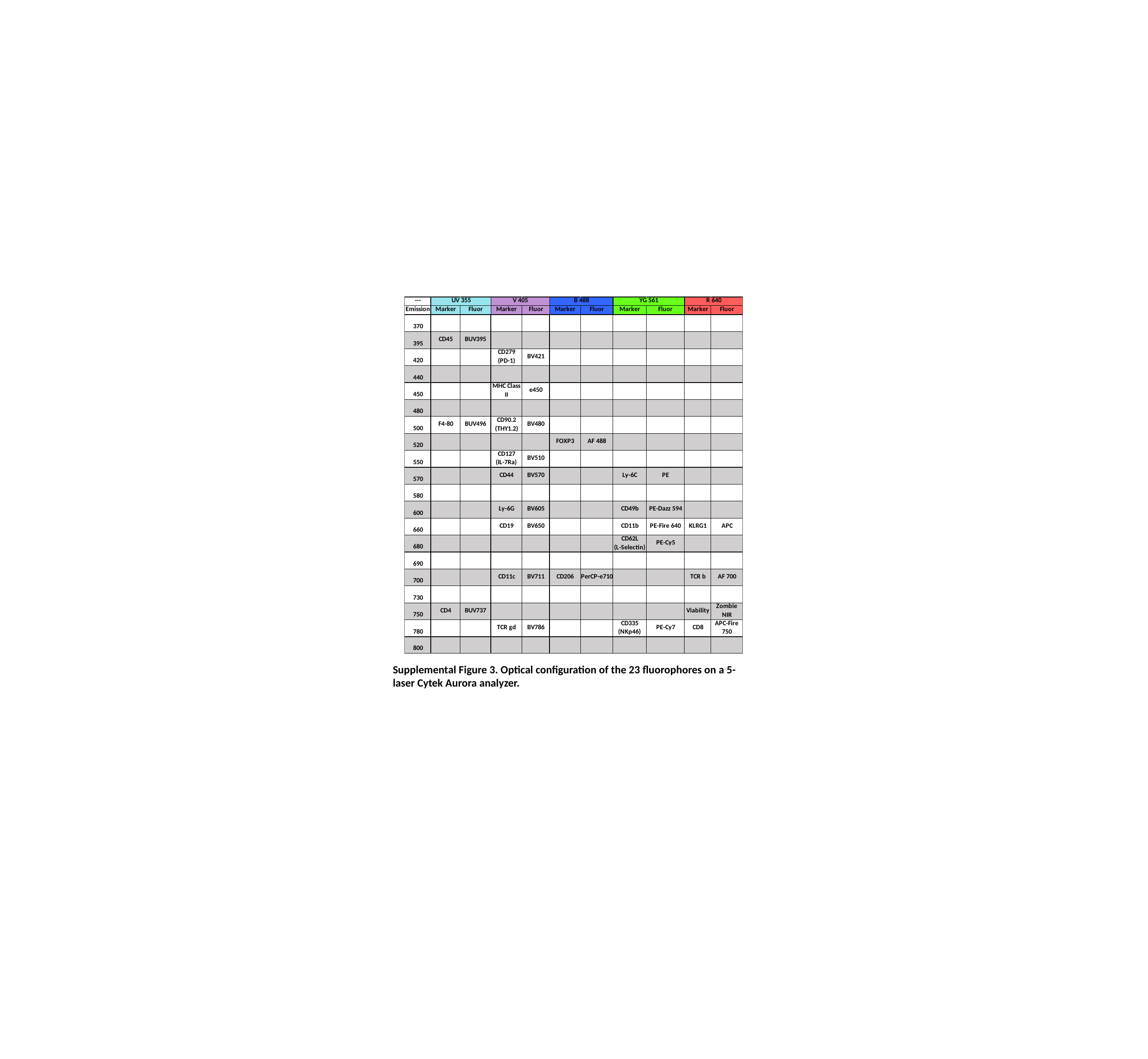

Supplemental Figure 3. Optical configuration of the 23 fluorophores on a 5-laser Cytek Aurora analyzer.

## Slide 4
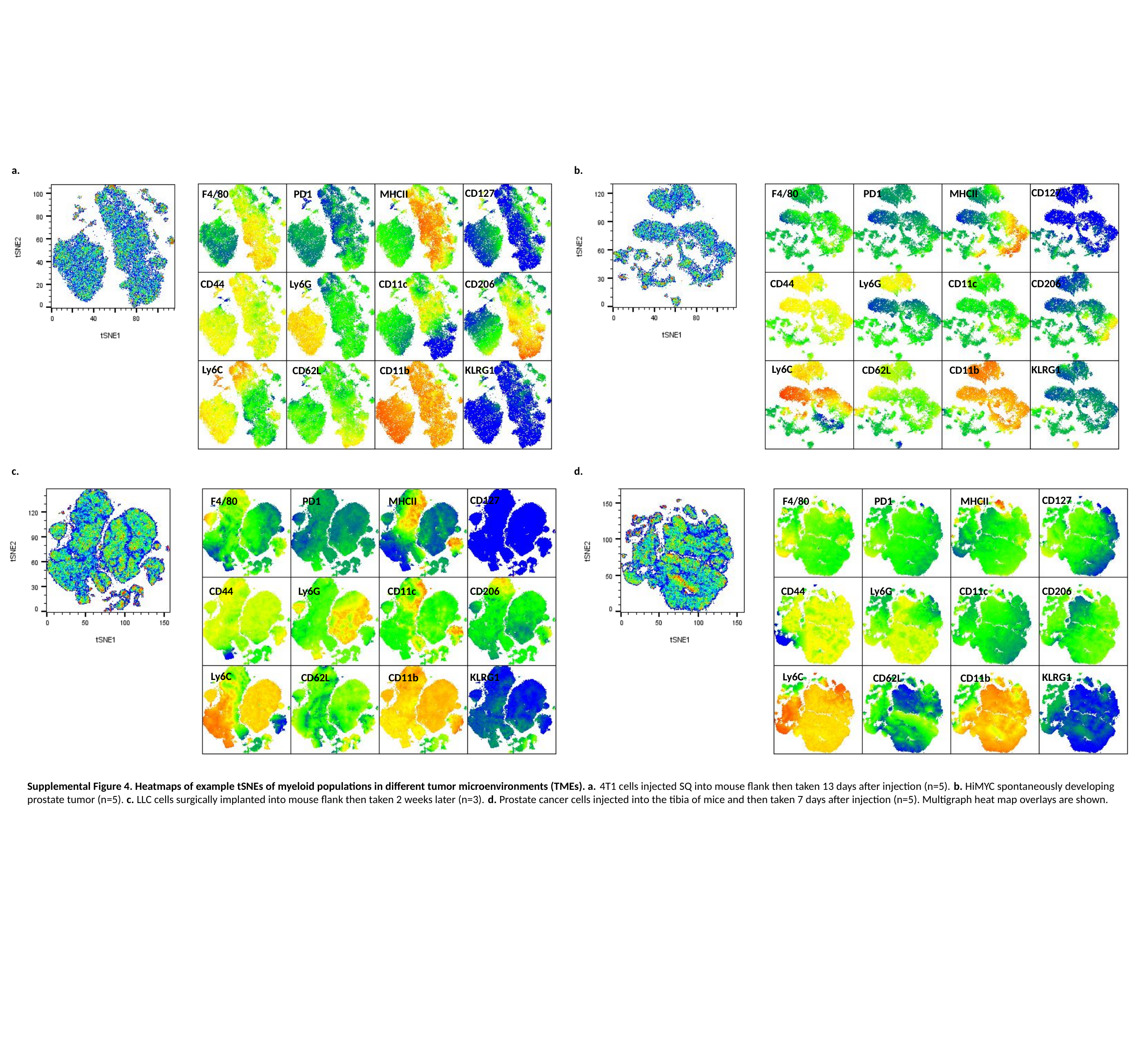

a.
b.
CD127
CD127
F4/80
PD1
MHCII
F4/80
PD1
MHCII
CD11c
CD206
CD44
Ly6G
CD11c
CD206
CD44
Ly6G
Ly6C
Ly6C
KLRG1
KLRG1
CD11b
CD62L
CD11b
CD62L
c.
d.
CD127
CD127
F4/80
PD1
MHCII
F4/80
PD1
MHCII
CD11c
CD206
CD44
Ly6G
CD11c
CD206
CD44
Ly6G
Ly6C
Ly6C
KLRG1
KLRG1
CD11b
CD62L
CD11b
CD62L
Supplemental Figure 4. Heatmaps of example tSNEs of myeloid populations in different tumor microenvironments (TMEs). a. 4T1 cells injected SQ into mouse flank then taken 13 days after injection (n=5). b. HiMYC spontaneously developing prostate tumor (n=5). c. LLC cells surgically implanted into mouse flank then taken 2 weeks later (n=3). d. Prostate cancer cells injected into the tibia of mice and then taken 7 days after injection (n=5). Multigraph heat map overlays are shown.

## Slide 5
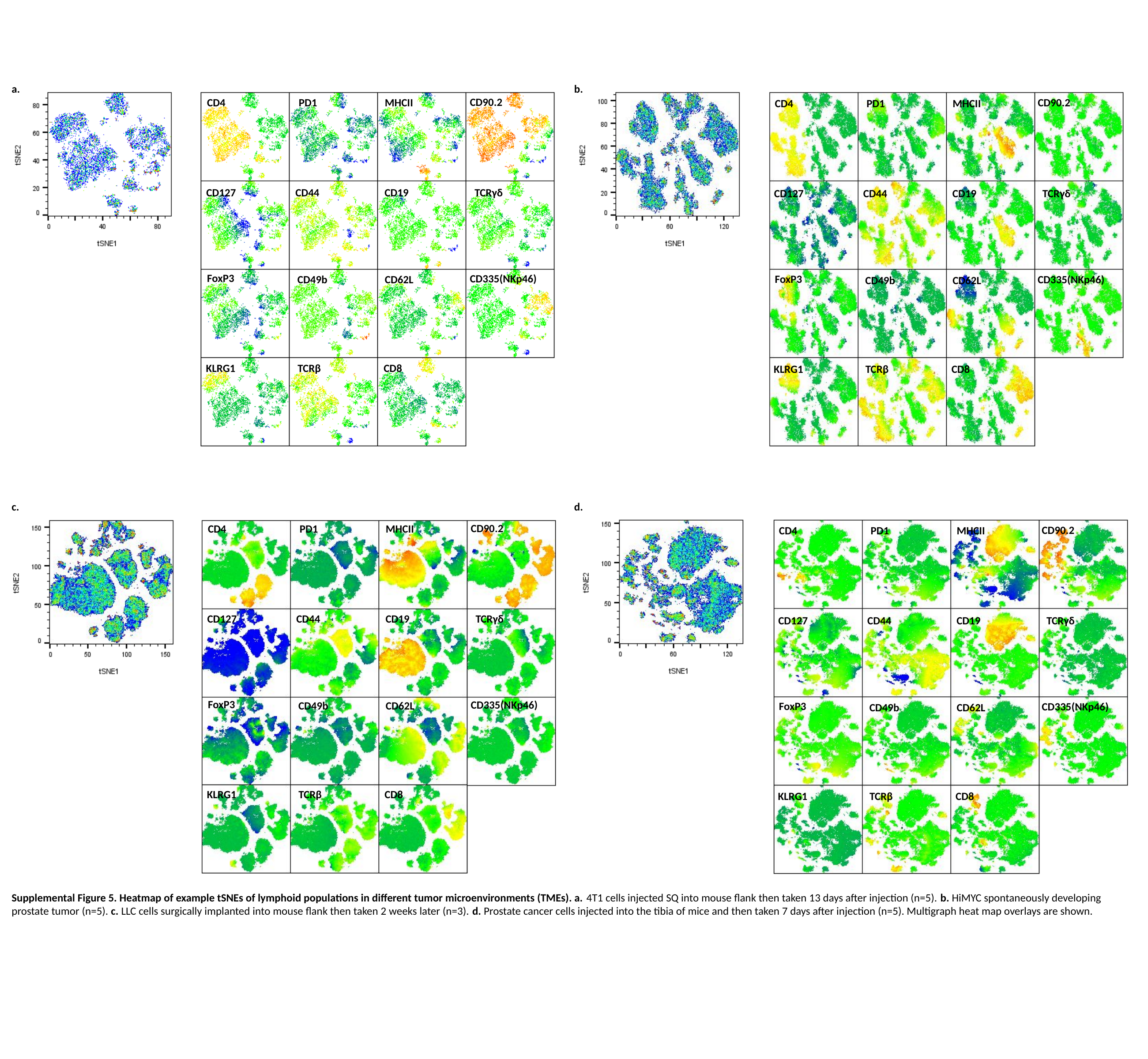

a.
b.
CD90.2
CD4
PD1
MHCII
CD90.2
CD4
PD1
MHCII
CD19
TCRγδ
CD127
CD44
CD19
TCRγδ
CD127
CD44
FoxP3
CD335(NKp46)
FoxP3
CD62L
CD335(NKp46)
CD49b
CD62L
CD49b
KLRG1
TCRβ
CD8
KLRG1
TCRβ
CD8
c.
d.
CD90.2
CD4
PD1
MHCII
CD90.2
CD4
PD1
MHCII
CD19
TCRγδ
CD127
CD44
CD19
TCRγδ
CD127
CD44
FoxP3
CD335(NKp46)
CD62L
CD49b
FoxP3
CD335(NKp46)
CD62L
CD49b
KLRG1
TCRβ
CD8
KLRG1
TCRβ
CD8
Supplemental Figure 5. Heatmap of example tSNEs of lymphoid populations in different tumor microenvironments (TMEs). a. 4T1 cells injected SQ into mouse flank then taken 13 days after injection (n=5). b. HiMYC spontaneously developing prostate tumor (n=5). c. LLC cells surgically implanted into mouse flank then taken 2 weeks later (n=3). d. Prostate cancer cells injected into the tibia of mice and then taken 7 days after injection (n=5). Multigraph heat map overlays are shown.

## Slide 6
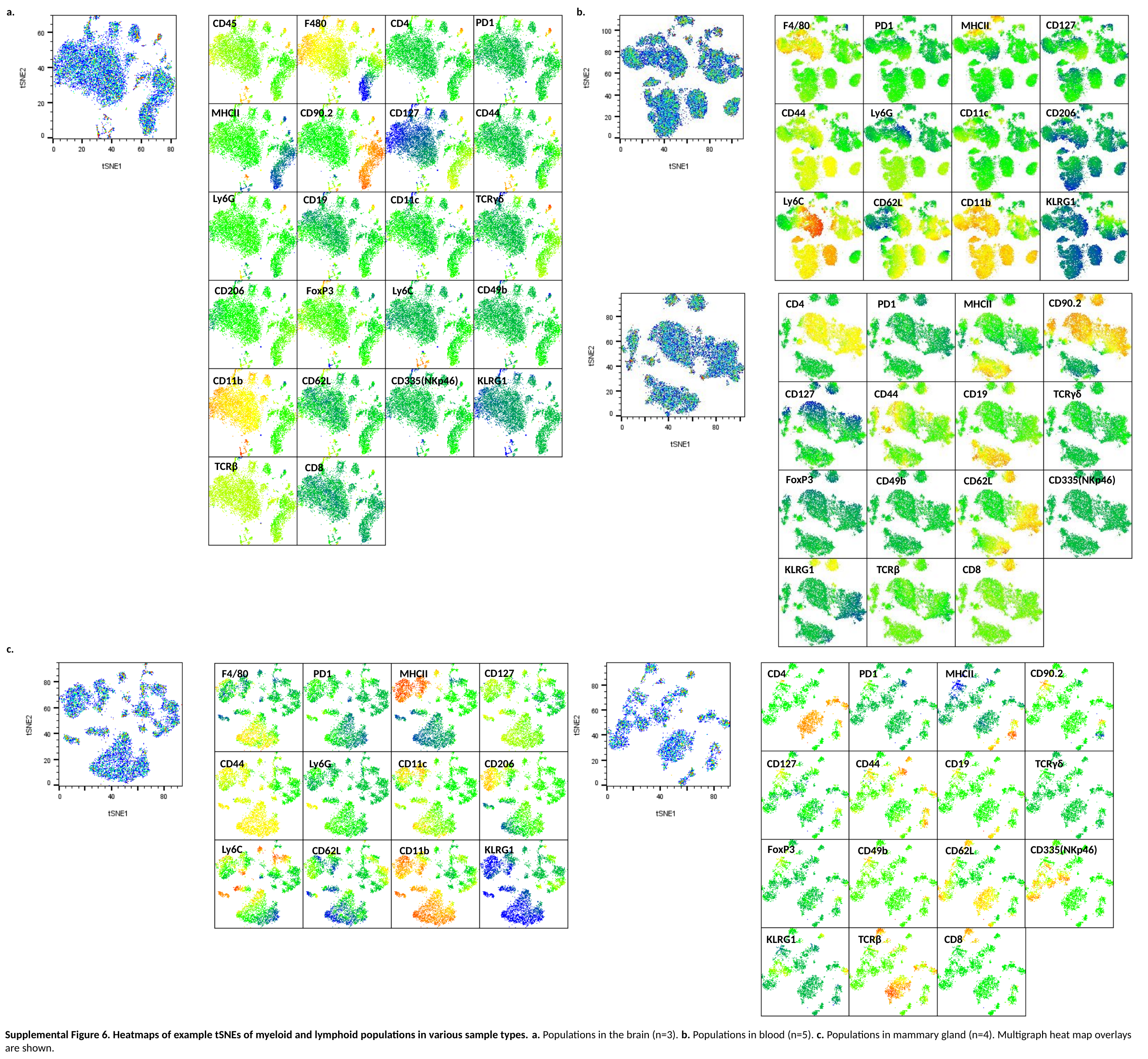

a.
b.
PD1
CD45
F480
CD4
CD127
F4/80
PD1
MHCII
CD11c
CD206
CD44
Ly6G
CD127
CD44
MHCII
CD90.2
Ly6G
TCRγδ
CD11c
CD19
Ly6C
KLRG1
CD11b
CD62L
CD49b
CD206
FoxP3
Ly6C
CD90.2
CD4
PD1
MHCII
CD335(NKp46)
KLRG1
CD11b
CD62L
CD19
TCRγδ
CD127
CD44
TCRβ
CD8
FoxP3
CD335(NKp46)
CD62L
CD49b
KLRG1
TCRβ
CD8
c.
CD127
CD90.2
F4/80
PD1
MHCII
CD4
PD1
MHCII
CD11c
CD206
CD19
TCRγδ
CD44
Ly6G
CD127
CD44
Ly6C
FoxP3
KLRG1
CD335(NKp46)
CD11b
CD62L
CD62L
CD49b
KLRG1
TCRβ
CD8
Supplemental Figure 6. Heatmaps of example tSNEs of myeloid and lymphoid populations in various sample types. a. Populations in the brain (n=3). b. Populations in blood (n=5). c. Populations in mammary gland (n=4). Multigraph heat map overlays are shown.

## Slide 7
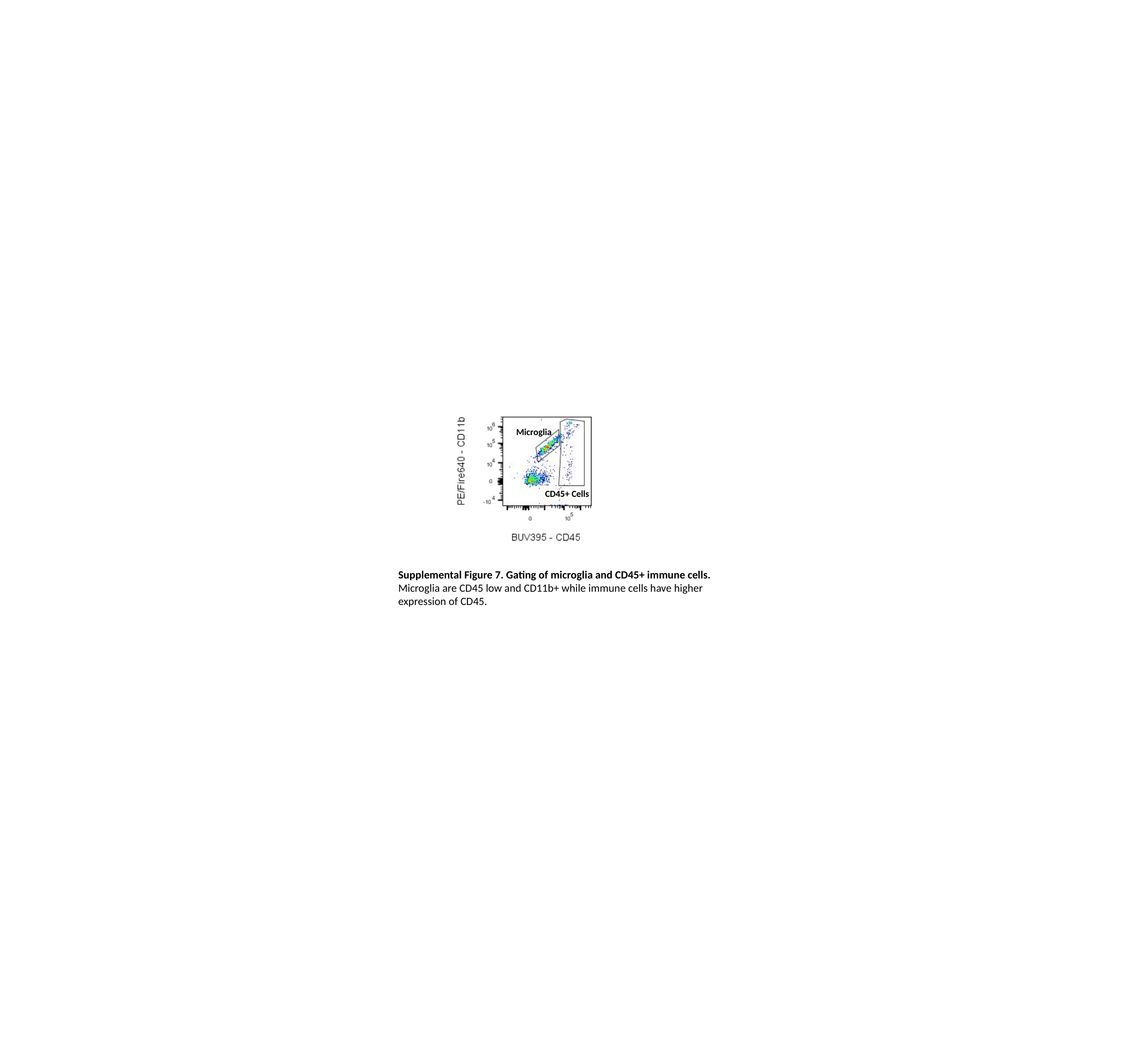

Microglia
CD45+ Cells
Supplemental Figure 7. Gating of microglia and CD45+ immune cells. Microglia are CD45 low and CD11b+ while immune cells have higher expression of CD45.

## Slide 8
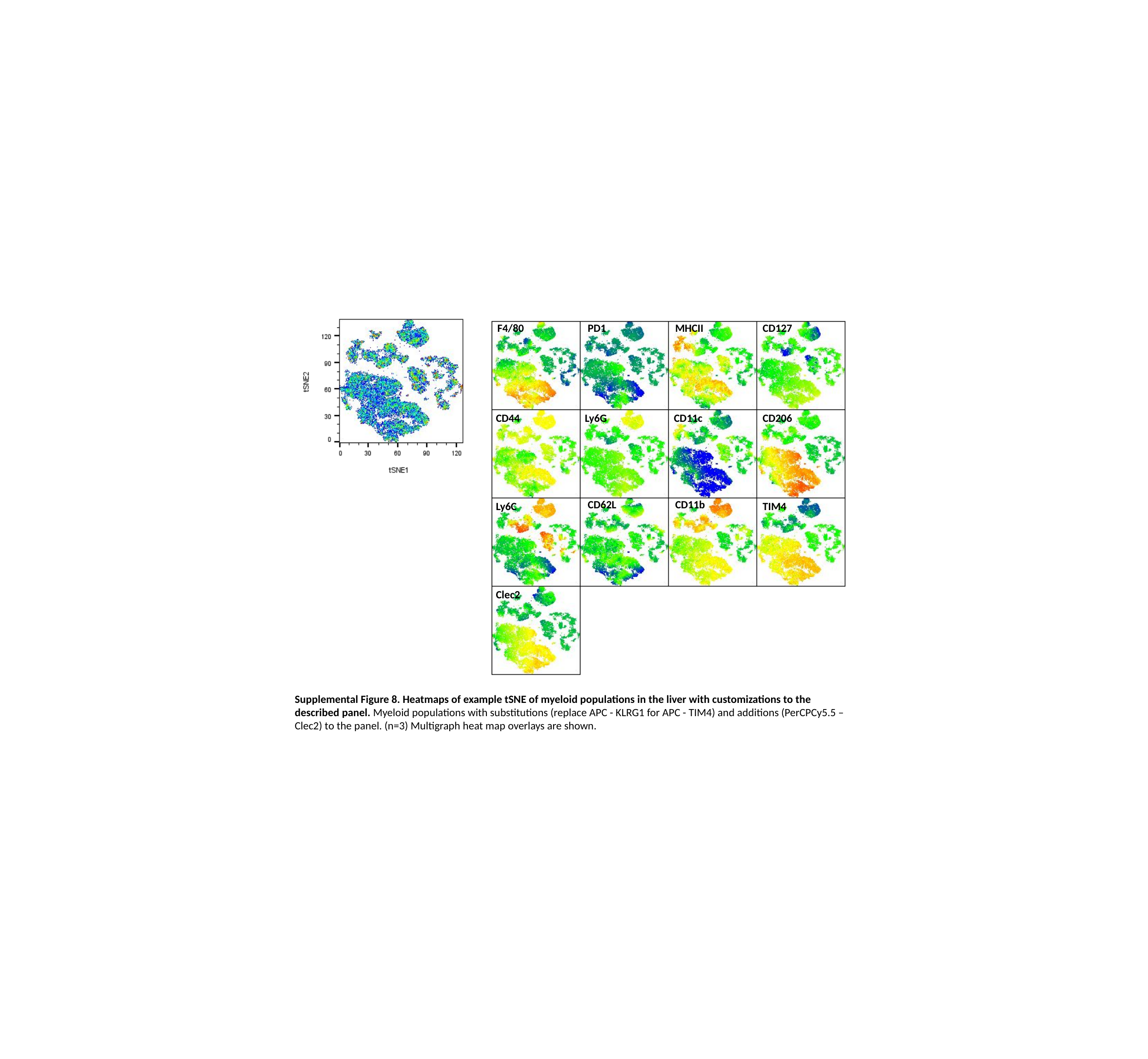

F4/80
CD127
MHCII
PD1
CD206
CD44
Ly6G
CD11c
CD11b
CD62L
Ly6C
TIM4
Clec2
Supplemental Figure 8. Heatmaps of example tSNE of myeloid populations in the liver with customizations to the described panel. Myeloid populations with substitutions (replace APC - KLRG1 for APC - TIM4) and additions (PerCPCy5.5 – Clec2) to the panel. (n=3) Multigraph heat map overlays are shown.

## Slide 9
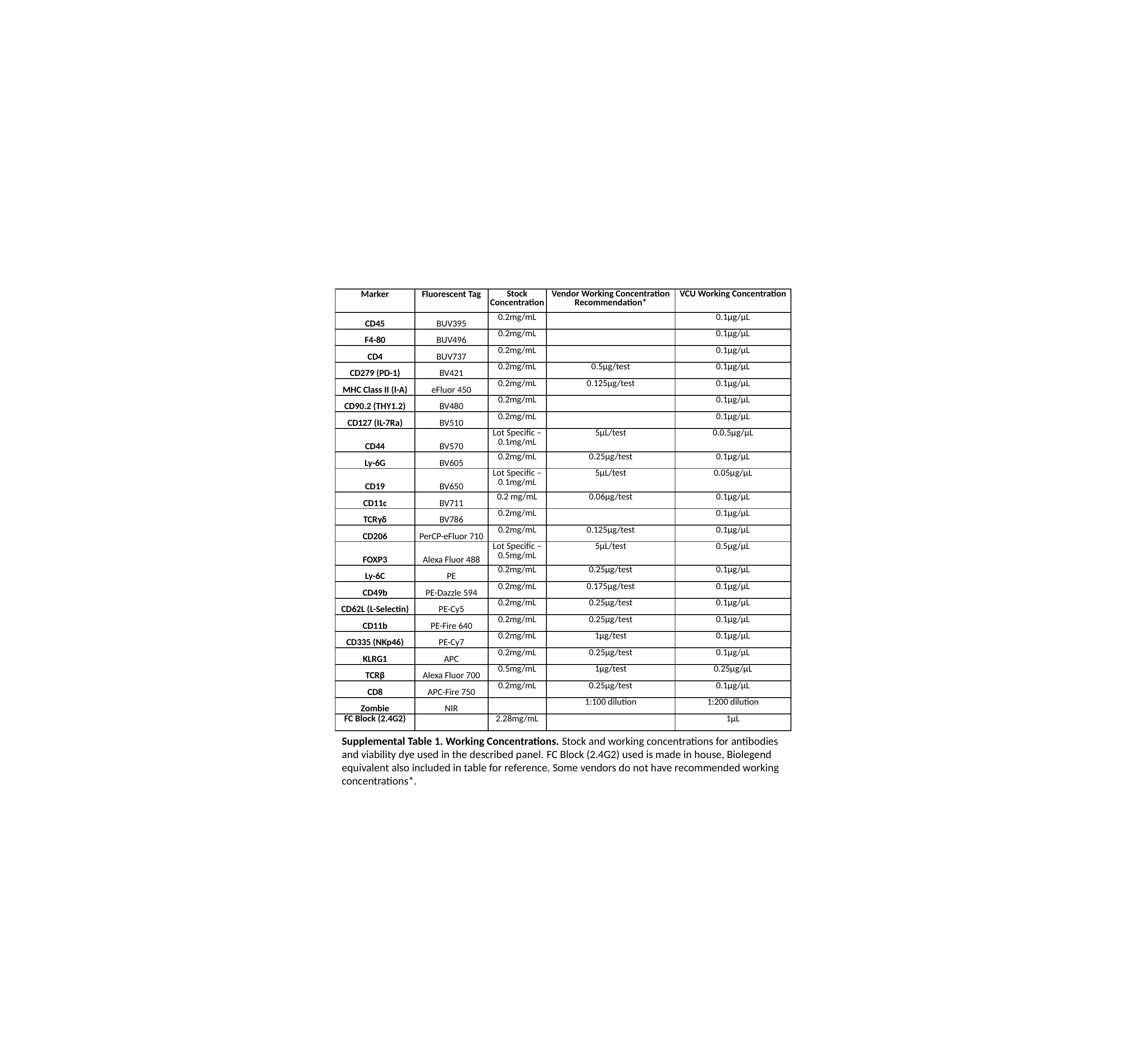

| Marker | Fluorescent Tag | Stock Concentration | Vendor Working Concentration Recommendation\* | VCU Working Concentration |
| --- | --- | --- | --- | --- |
| CD45 | BUV395 | 0.2mg/mL | | 0.1µg/µL |
| F4-80 | BUV496 | 0.2mg/mL | | 0.1µg/µL |
| CD4 | BUV737 | 0.2mg/mL | | 0.1µg/µL |
| CD279 (PD-1) | BV421 | 0.2mg/mL | 0.5µg/test | 0.1µg/µL |
| MHC Class II (I-A) | eFluor 450 | 0.2mg/mL | 0.125µg/test | 0.1µg/µL |
| CD90.2 (THY1.2) | BV480 | 0.2mg/mL | | 0.1µg/µL |
| CD127 (IL-7Ra) | BV510 | 0.2mg/mL | | 0.1µg/µL |
| CD44 | BV570 | Lot Specific – 0.1mg/mL | 5µL/test | 0.0.5µg/µL |
| Ly-6G | BV605 | 0.2mg/mL | 0.25µg/test | 0.1µg/µL |
| CD19 | BV650 | Lot Specific – 0.1mg/mL | 5µL/test | 0.05µg/µL |
| CD11c | BV711 | 0.2 mg/mL | 0.06µg/test | 0.1µg/µL |
| TCRγδ | BV786 | 0.2mg/mL | | 0.1µg/µL |
| CD206 | PerCP-eFluor 710 | 0.2mg/mL | 0.125µg/test | 0.1µg/µL |
| FOXP3 | Alexa Fluor 488 | Lot Specific – 0.5mg/mL | 5µL/test | 0.5µg/µL |
| Ly-6C | PE | 0.2mg/mL | 0.25µg/test | 0.1µg/µL |
| CD49b | PE-Dazzle 594 | 0.2mg/mL | 0.175µg/test | 0.1µg/µL |
| CD62L (L-Selectin) | PE-Cy5 | 0.2mg/mL | 0.25µg/test | 0.1µg/µL |
| CD11b | PE-Fire 640 | 0.2mg/mL | 0.25µg/test | 0.1µg/µL |
| CD335 (NKp46) | PE-Cy7 | 0.2mg/mL | 1µg/test | 0.1µg/µL |
| KLRG1 | APC | 0.2mg/mL | 0.25µg/test | 0.1µg/µL |
| TCRβ | Alexa Fluor 700 | 0.5mg/mL | 1µg/test | 0.25µg/µL |
| CD8 | APC-Fire 750 | 0.2mg/mL | 0.25µg/test | 0.1µg/µL |
| Zombie | NIR | | 1:100 dilution | 1:200 dilution |
| FC Block (2.4G2) | | 2.28mg/mL | | 1µL |
Supplemental Table 1. Working Concentrations. Stock and working concentrations for antibodies and viability dye used in the described panel. FC Block (2.4G2) used is made in house, Biolegend equivalent also included in table for reference. Some vendors do not have recommended working concentrations*.
